# A novel method for interpreting survival analysis data: description and test on three major clinical trials on cardiovascular prevention

**DOI:** 10.1186/s13063-020-04511-y

**Published:** 2020-06-26

**Authors:** Alessandro Mengozzi, Domenico Tricò, Andrea Natali

**Affiliations:** 1 Department of Clinical and Experimental Medicine, University of Pisa, Via Savi 10, Pisa, Italy; 2 Institute of Life Sciences, Sant’Anna School of Advanced Studies, Pisa, Italy

**Keywords:** Survival analysis, Clinical trials, Hazard ratio, Kaplan-Meier, Non-parametric

## Abstract

**Background:**

Major results of randomized clinical trials on cardiovascular prevention are currently provided in terms of relative or absolute risk reductions, including also the number needed to treat (NNT), incorrectly implying that a treatment might prevent the occurrence of the outcome/s under investigation. Provided that these results are based on survival analysis, the primary measure of which is time-to-the outcome and not the outcome itself, we sought an alternative method to describe, analyse and interpret clinical trial results consistent with this assumption, so as to better define qualitative and quantitative heterogeneity of various therapeutic strategies in terms of their effects and costs.

**Methods:**

The original Kaplan-Meier graphs of three major positive cardiovascular prevention trials (PROVE-IT, LIFE and HOPE) were captured from the PDF images of the article and then digitalized. We calculated the difference between the placebo and active treatment curves and plotted it as a function of time to describe the event-free time gain (*Time-Gain*) produced by the active treatment. By calculating the exposure to the active treatment in terms of months (MoT) as a function of time and dividing it for the corresponding time-dependent number of event-free years gained (i.e. months/12), we described the kinetics of the pharmaco-economic index MoT/y^+^. The same procedure was repeated replacing MoT with the actual number of patients being treated at each time point as a function of time to obtain the NNT to gain 1 event-free year (NNT/y^+^) curve.

**Results:**

The *Time-Gain* curves depict the kinetics of the treatment-related effect over time and possess the peculiar feature of being smooth and accurately fitted by second-order polynomial functions (a*time^2^ + b*time); similarly, also the MoT/y^+^ and NNT/y^+^ curves can be accurately fitted by power functions (a*time^*b*^).

These curves and indices allow to fully appreciate the quantitative and qualitative heterogeneity, both in terms of effects and costs, of the different therapeutic strategies adopted in the three trials.

**Conclusions:**

With our novel method, by exploiting original Kaplan-Meier curves from three major clinical trials on cardiovascular prevention, we generate new information on the actual consequences of choosing a therapeutic strategy vs another, thus ultimately providing the clinical gain in terms of time-dependent functions. Accurately assessing clinically and economic meaningful results from any intervention trial reporting positive results through this approach, facilitates objective comparisons and increases reliability in predicting survival among the various therapeutic options provided.

**Trial registration:**

PROVE-IT (Pravastatin or Atorvastatin Evaluation and Infection Therapy (TIMI22), Clinical trial registration number: NCT00382460, date of registration: September 29, 2006, study start date: November 2000).

LIFE (Losartan Intervention For Endpoint Reduction in Hypertension (LIFE) Study, Clinical trial registration number: NCT00338260, date of registration: June 20, 2006, study start date: June 1995).

HOPE (Heart Outcomes Prevention Evaluation; we could not find Clinical trial registration number and date of registration).

## Introduction

The standard presentation of randomized clinical trials (RCT) comparing treatment strategies is based on hazard ratios (HR) and eventually on the number needed to treat (NNT). When a strategy is shown to be superior, the message conveyed is that it reduces the risk to develop (i.e. it prevents) the outcome under consideration. Assuming that in most studies the primary outcome variable is the time-to-the-event and not the event itself, the message may be misleading. In addition, the true benefit of any intervention, when effective, consists of the longer length of event-free time experienced by the cohort when compared to that on the alternative treatment. Extending event-free life is not equivalent to prevent (avoid) the occurrence of the event. This is particularly evident when the outcome is unavoidable like death, but it also holds true for any other event that is likely to occur in the specific population, namely like cardiovascular (CV) events in high-risk groups.

The need to move beyond the HR, as highlighted in recent oncology literature [1], is not only based on the elusive clinical implications of the HR itself, but also on the formal requirement that the ratio of the two hazard functions is constant over time, which is seldom verified and not always self-evident. Median survival [2], accelerated failure-time model [3, 4] and quantile regression [5] have been proposed as alternatives to overcome these limitations [1, 2, 6]; however, they require that at least 50% of the sample population experienced the outcome of interest, and in case of CV prevention trials, this is seldom the case. Projecting half-lives beyond the duration of the trial is an alternative; however, it relies on the assumption that the slope of the survival curve over time follows a predictable function, which has been demonstrated to be incorrect by Meier-Kriesche et al [7]. These authors suggested that the difference in the area under the Kaplan-Meier (KM) curves of the two treatment groups can provide a better and more reliable estimate for the treatment effect, especially for short-terms studies. The need for alternatives to the HR based on the analysis of time, particularly for non-inferiority trials, has been elegantly highlighted in recent paper by Uno et al. [8] who purposefully stated that “... the patients’ exposure times are more clinically important than the observed number of event” and it is underscored that unlike proportional hazards, the analysis of time has also the advantage of not requiring specific assumptions. Lytsy et al. [9] proposed to adopt a non-parametric description of the time course of the delay of events. This parameter has the merit of focusing on the time-to-the-event rather than on the event itself, but has the flaw of not considering the exposures’ times; therefore, it remains essentially descriptive.

Pharmaco-economy is a second perspective to be taken into account. Focusing on time-to-the-event is instrumental for accurate cost-benefit analysis allowing estimates over time and, eventually, their prediction beyond the duration of the study, which becomes especially relevant when comparing novel and more expensive treatments to standard ones.

Finally, a third important issue is the comparison among studies. To rely only on crude numbers such as HR and NNT, especially for studies with different lengths, might not only be misleading but, most importantly, does not convey all the information generated by all data collected throughout the study.

Our objective was to extract all the information contained in a clinical trial’s KM curves. To this aim, we approached the trial as a biological experiment where an intervention is applied over time (exposure) in order to produce a response (event-free time gain), which might follow a specific kinetics. Subsequently, from these dose-response curves, we derived indices able to effectively describe the trial’s results in pharmaco-economic terms, allowing comparisons among different interventions.

To do so, we here present a novel method that by exploiting KM curves enables a clinically meaningful representation of the major results generated by any positive clinical trial. The method, named PISA (Pragmatic Interpretation of Survival Analysis), is described in detail and tested on PROVE-IT [10], LIFE [11] and HOPE [12], three major, heterogeneous and positive CV prevention clinical trials.

## Methods

### Trial selection

The criteria were as follows: (a) active treatment vs standard or head-to-head comparison, (b) superiority clearly demonstrated, (c) high-quality KM images, (d) CV outcomes, (e) studies lasting more than 2 years, (f) continuous treatment throughout the study, (g) population size greater than 4000.

### Data extraction

**Fig. 1:**
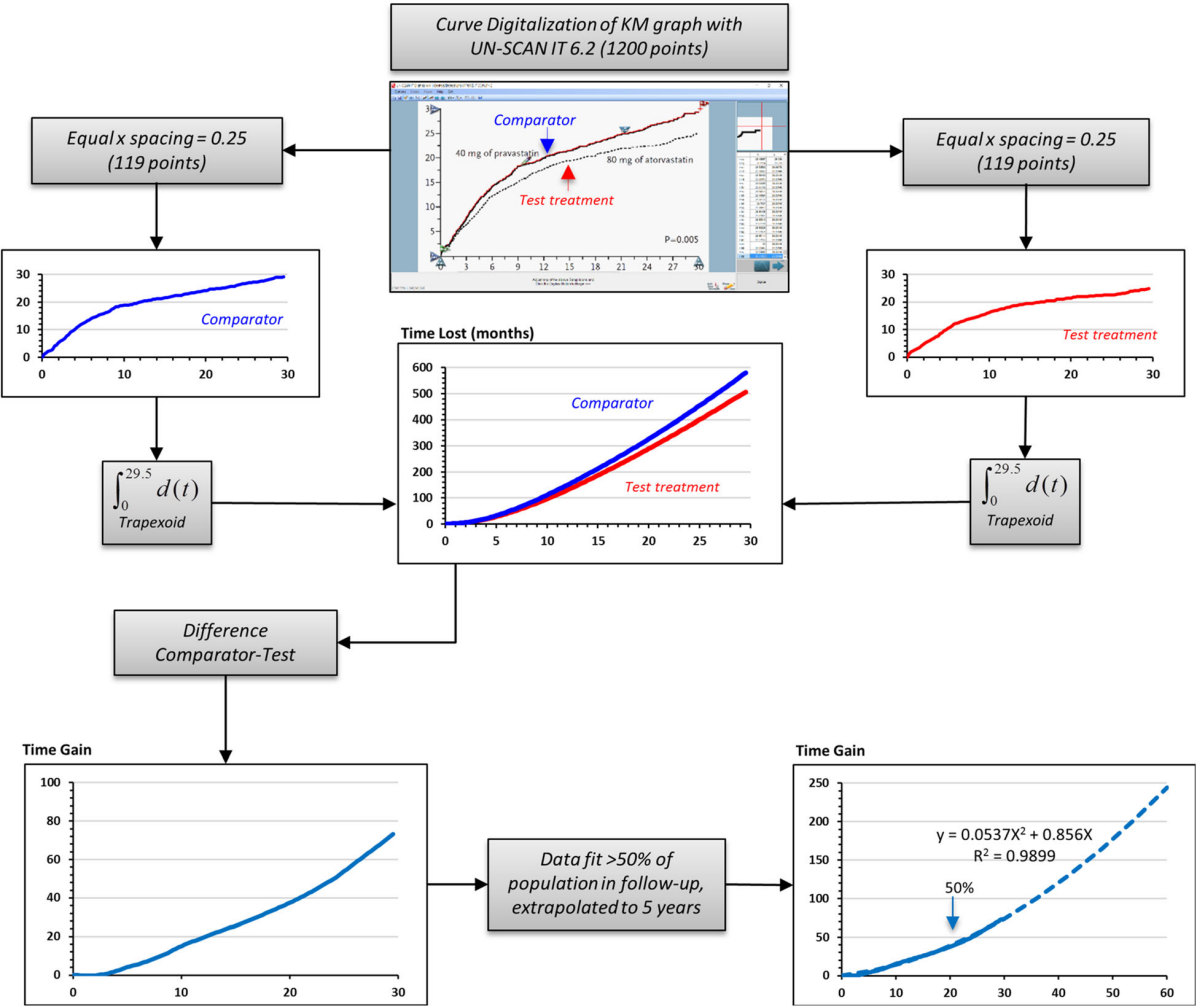
Graphic illustration of the PISA method. Original inverse Kaplan-Meier graphs were first captured from the PDF of the article as high-definition images (.png) and then converted into data using the UN-SCAN-IT Graph Digitalizer software. Digitalized data were visualized and misplaced points (< 0.1%) manually shifted or erased and replaced. Time (*x*-axis) spacing was then forced to 0.25 months, and when missing the incidence (*y*-axis), data was automatically interpolated with the linear method using the two closest points. The integral function of the inverse KM curves (Time-Lost) was obtained by applying piecewise integration using the trapezoid rule with equal *x* segments. Then, the Time-Gain curve was obtained from the difference between the integrals of placebo and active treatment, plotted as a function of time. Finally, we performed curve fitting on data up to the time when 50% of the total population was still being followed up

As illustrated in Fig. 1, original inverse KM graphs (i.e. indicating cumulative incidence instead of event-free survival) were first captured from the PDF of the article as high-definition images (.png) and then converted into data using the UN-SCAN-IT Graph Digitizer software (Silk Scientific, Inc. Orem, Utah USA). After several attempts, we selected the following software parameters for line following being the more efficient: (1) “point assignment”: midline of upper and lower surface of the curve; (2) “line follow algorithm”: sloped; (3) sampling: 1 scanner unit (which ranged from 0.027 to 0.081 months among the three studies). Digitalized data (408–1080 couples of time and % values) were visualized and misplaced points at visual inspection (< 0.1%) were manually shifted or erased and replaced. In order to provide homogeneous data sets, to be transferred in an ad hoc built spreadsheet for subsequent calculations, time (*x* axis) spacing was then forced to 0.25 months yielding sets of 120–266 couples and the few (< 1%) missing incidence (*y* axis) data were automatically interpolated with the linear method using the closest upper and lower points. To provide an internal validation, we tested the method through two different operators in four different days: the % of total variance attributable to the intra- and inter-observer variability was < 0.1%. We could not perform any external validation (i.e. accuracy of extracted data with respect to the original ones) since the original data were not available; however its accuracy can be visually appreciated from Supplementary Figure 1.

### Data calculation of the PISA approach

A full glossary with definitions and mathematical formulae is provided in Supplementary Figure 2.

- Time-Gain curves

To assess the benefit (event-free time gain) of the treatments, we drew the Time-Gain curves. First, we calculated the integrals of each inverse KM applying piecewise integration using the trapezoid rule with equal *x* segments (0.25 months) and then we plotted it as a function of time. This function (*Time-Lost*) represents the time course of the units of time (months) spent after the specific event has occurred that progressively accumulate during the follow-up in a group of 100 individuals at baseline. The difference of the placebo and active treatment Time-Lost curves (i.e. the difference between the two KM areas under the curve), also plotted as a function of time, describes the event-free time (*Time-Gain*) that is produced by the active treatment. Positive values indicate a protective effect of the active treatment vs control, whereas negative values represent a harmful effect (i.e. a loss of event-free time).

The Time-Gain curve, when the active treatment is effective (i.e. the two original inverse KM curves separate with time), has a peculiar characteristic: it follows a kinetics that can be accurately described by a second-order polynomial function forced to pass through the origin (a*time^2^ + b*time). This was verified also in other CV outcome clinical trials of heterogeneous durations in whom different types of treatments were effective like UKPDS-34, STENO-2, CIBIS-II, EMPA-REG OUTCOME, CANVAS, LEADER and SUSTAIN-6 (Supplementary Figure 3).

In order to facilitate comparisons, to avoid loss of time-dependent information and to attenuate the uncertainties introduced by the progressive reduction of the number of the subjects in follow-up, we exploited the peculiar kinetics of the curves to perform curve fitting on data. The interval on which the fit was performed was from time 0 to the time when 50% of the total population was still being followed or, when this was not possible (as in the HOPE trial, where data on the percentage of population in follow-up was not available) at 50% of the length of follow-up. Data fit, performed using standard regression procedures, was then accepted only for regression coefficient values > 0.95. The resulting equations were used to generate Time-Gain f50% curves and the math coefficients describing all studies; for the PROVE-IT (the shorter of the tree), the fit was used to extrapolate data beyond the actual duration of the study.

b) Months of treatment per event-free years (MoT/y^+^).

The cost effectiveness of any treatment results from the ratio between drug exposure and clinical benefit and is a function of time. Accordingly, we generated the pharmaco-economic index MoT/y^+^ (Fig. 3), which represent the number of months of treatment of the entire cohort that is necessary to gain 1 year of event-free life at any given time during the trial. This was obtained as the exposure to the active treatment, expressed as total months of treatment and calculated as the integral (area under the curve) of the percent of subjects without the event (i.e. KM survival curves), divided for the corresponding time-dependent number of years (i.e. months/12) gained as a consequence of the treatment. To avoid loss of information, as described in section “Time-Gain curves”, we also calculated the MoT/y^+^f50%, where the values of years gained were assessed through the Time-Gain f50% function instead of the actual Time-Gain curve.

The MoT/y^+^f50% curve is initially very noisy since the denominator is extremely small; however, when the value of 6 months of Time-Gain is achieved, it follows very closely a power kinetics (a*time^*b*^). To maximize the precision of the fit, the interval on which it was performed was therefore from when the value of 6 months gained was reached to the end of the follow-up. In order to allow homogeneous comparisons among the trials, the equation obtained through this curve fitting (eMoT/y^+^) was used to calculate the data at fixed time points (eMoT/y^+^@2 years and eMoT/y^+^@6 years).

c) Number needed to treat per event-free year (NNT/y^+^).

Since the NNT is an index extensively used in the medical literature, and also easily understood by the medical community, we generated another pharmaco-economic index, the NNT/y^+^, that represents the number of subjects who need to be treated to gain 1 year of event-free time at any given time during the trial.

It was calculated with the same procedure adopted for the MoT/y^+^, replacing the MoT with the actual number of patients being treated at each time point (obtained by dividing the MoT for the time of the study) to obtain the NNT to gain one event-free year (NNT/y^+^f50%) and the corresponding eNNT/y^+^@2 years and eNNT/y^+^@6 years indices.

## Results

### The Time-Gain curves

**Fig. 2:**
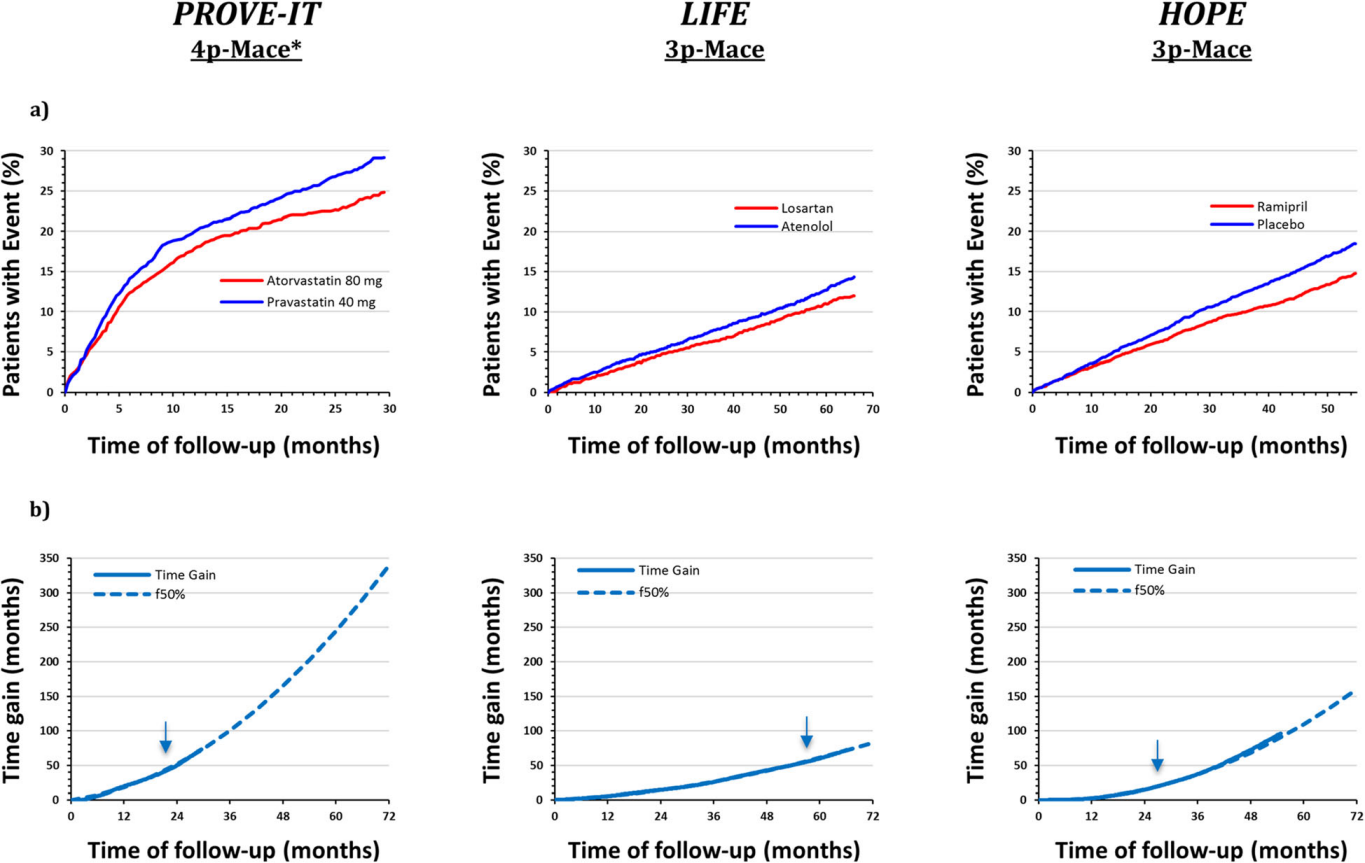
Incidence plots and Time-Gain curves for PROVE-IT (4p-MACE), LIFE (3p-MACE) and HOPE (3p-MACE) trials. 4p-MACE* outcome: death from any cause, myocardial infarction, documented unstable angina requiring rehospitalization, revascularization with either percutaneous coronary intervention or coronary-artery bypass grafting—if these procedures were performed at least 30 days after randomization—and stroke; 3p-MACE outcome: cardiovascular death, non-fatal stroke and non-fatal myocardial infarction), **a** Incidence plots in the PROVE-IT (*blue line: pravastatin 40 mg, red line: atorvastatin 80 mg*), LIFE (*blue line: atenolol, red line: losartan*) and HOPE (*blue line: placebo, red line: ramipril*) studies. **b** Observed Time-Gain curves (*continuous line*) throughout the duration of the three studies and fitted Time-Gain curves *(f50%*; *dotted lines*) with the extrapolation beyond time at which less than 50% of the cohort was in follow-up (*indicated by arrows*) for PROVE-IT and LIFE trials. For the HOPE trial (due to the absence of data of population in follow-up), the fit and the extrapolation were performed beyond half the duration of the trial (*indicated by arrows*)

The length of follow-up, the absolute rate and the kinetics of the event accrual as well as the effect of the intervention were rather different among the three clinical trials used to test the PISA method (Fig. 2). The event accumulation was not linear over time and five- to threefold greater in the PROVE-IT study with respect to LIFE and HOPE trials. The time necessary for the effect of the treatment to emerge (i.e. before the KM curves start to diverge) was longer for HOPE, but only in this study the separation of the curves showed a stable trend to increase over time. Despite this heterogeneity, the calculated Time-Gain curves were able to describe the kinetics of the treatment-related benefit over time and showed constant characteristics. They were all smooth and could be accurately described—through non-linear regression—by a second-order polynomial function. The accuracy of this fit is demonstrated by the high regression coefficient values calculated within the interval used for the fit (*R*^*2*^*value 0-t*_*50%*_) (Table 1). The ability of the fit in predicting the actual Time-Gain well beyond the time window used for its calculation is particularly evident in HOPE and PROVE-IT, in which the *t*_50%_ fall at 50 and 70%, respectively, of the whole study duration (comparison of continuous and dotted lines in Fig. 2 and comparisons of Time-Gain *R*^2^ values *0-t*_*50%*_ vs *0-T* in Table 1). The heterogeneity of true clinical benefit produced by the three interventions clearly emerges from the comparisons of Time-Gain@6 years, being in HOPE double and in PROVE-IT fourfold than what is observed in LIFE (Table 1), a difference, which cannot be entirely appreciated by comparing usual indices of intervention efficacy (relative risk and absolute risk reduction).

**Table 1:** Trials (PROVE-IT [10]^,^ LIFE [11]^,^ HOPE [12]) data and observed or estimated indices of treatment effect according to the PISA method

	**PROVE-IT**	**LIFE**	**HOPE**
**Duration/*t***_**50%**_**(months)**	**30/21**	**66/58**	**55/*27***
**Outcome**	**4p-MACE**	**3p-MACE**	**3p-MACE**
**Rate comparator** (%/year)	**11.6**	**2.8**	**3.7**
**RRR [95%CI]** (%)	**16 [5–26]**	**13 [2–23]**	**22 [14–30]**
**ARR** (%)	**4.65**	**1.95**	**3.71**
**Time-Gain@2 years** (m)	**49.9**	**14.5**	**14.8**
**Time-Gain@T**	**73.2**	**72.0**	**96.4**
*f50% 2nd order coef.*	*0.0537*	*0.0116*	*0.0335*
*f50% 1st order coef.*	*0.856*	*0.3186*	*−0.186*
*R*^*2*^*value 0-t*_*50*_	*0.9899*	*0.9991*	*0.9995*
*R*^*2*^*value 0-T*	*0.9970*	*0.9990*	*0.9994*
*Time-Gain f50%@2 years (m)*	*51.5*	*14.3*	*14.8*
*Time-Gain f50%@6 years (m)*	*340.0*	*83.1*	*160.3*
**MoT/y**^**+**^**@2 years**	**487**	**1946**	**1871**
**MoT/y**^**+**^**@T**	**400.3**	**1032.9**	**692.2**
*Fit coef.*	*2757*	*11,459*	*109,635*
*Fit exp.*	*−0.554*	*−0.562*	*− 1.278*
*R*^*2*^*value 0-T*	*0.9579*	*0.9686*	*0.9990*
*eMoT/y*^*+*^*@2 years*	*474*	*1920*	*1888*
*eMoT/y*^*+*^*@6 years*	*258*	*1036*	*464*
**NNT/y**^**+**^**@2 years**	**20.3**	**81.1**	**78.0**
**NNT/y**^**+**^**@T**	**13.6**	**15.7**	**11.5**
*Fit coef.*	*2757*	*11,459*	*109,635*
*Fit exp.*	*−1.554*	*−1.562*	*−2.278*
*R*^*2*^*value*	*0.9901*	*0.9964*	*0.9994*
*eNNT/y*^*+*^*@2 years*	*19.8*	*80.0*	*78.6*
*eNNT/y*^*+*^*@6 years*	*3.6*	*12.7*	*6.4*

Estimated indices and functions are presented in italics. *RRR* relative risk reduction, *ARR* absolute risk reduction, *T* end of the study, *Time-Gain@T* the value of even-free months accumulated at the end of the study, *f50%* fit performed at 50% of the study follow-up length, *MoT/y*^*+*^ months of treatment per event-free years, *eMoT/y*^*+*^ MoT/y^+^ estimated through curve fitting performed on time interval from 6 months to the end of the follow-up, *NNT/y*^*+*^ number needed to treat per event-free years, *eNNT/y*^*+*^ NNT/y^+^ estimated through curve fitting performed on time interval from 6 months to the end of the follow-up

### The MoT/y^+^ curves

**Fig. 3:**
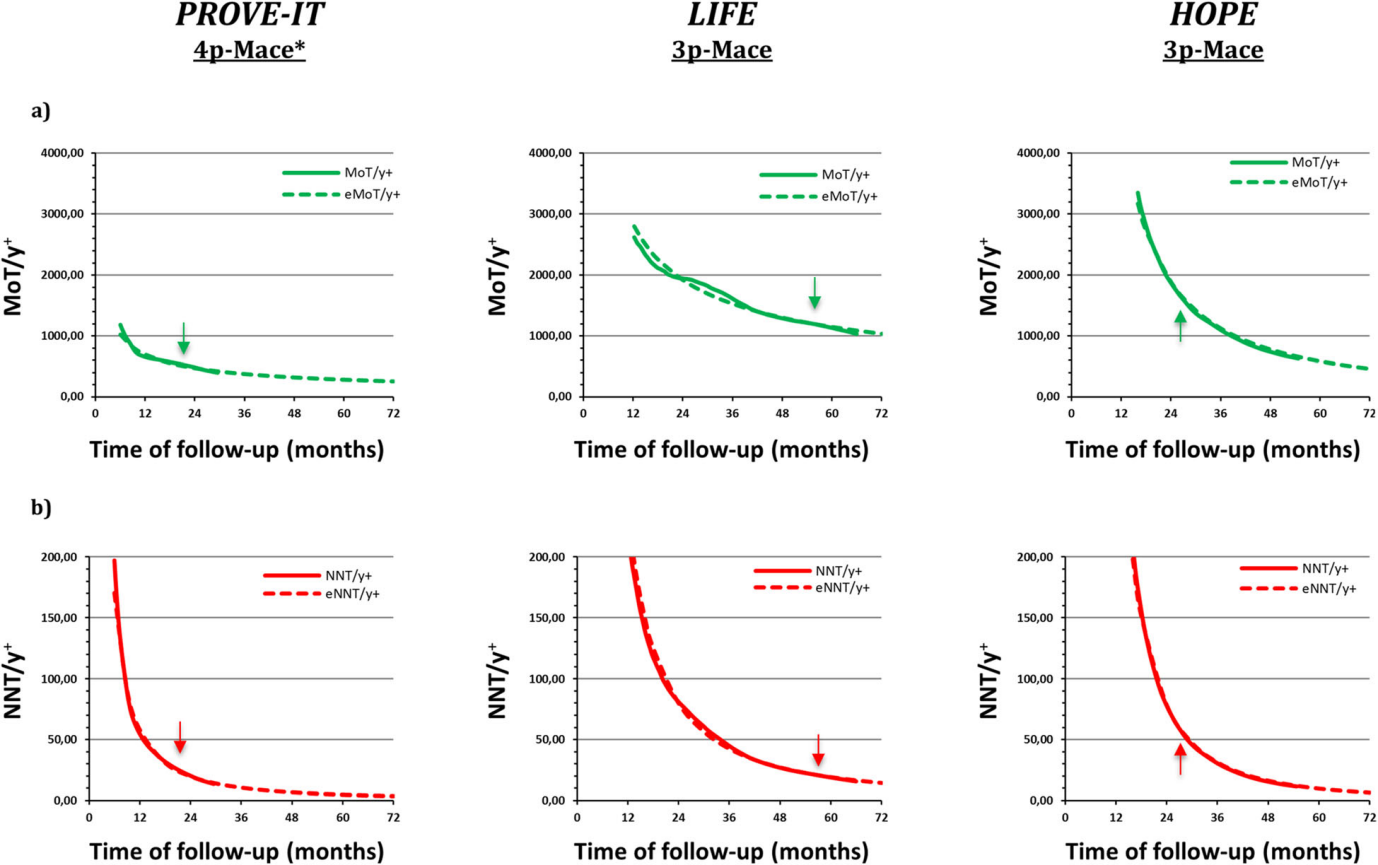
Pharmaco-economic indices MoT/y^+^ and NNT/y^+^ for PROVE-IT (4p-MACE*), LIFE (3p-MACE) and HOPE (3p-MACE) trials. **a** Observed (*continuous green lines*) Months of treatment per 1 year gained (MoT/y^+^) curves throughout the three trial and estimated MoT/y^+^*(eMoT/y*^*+*^*; dotted green lines*) up to time 72 months. The arrows show the time at which the 50% of the cohort was in follow-up for PROVE-IT and LIFE trial and the 50% of the duration of the study for the HOPE trial. **b** Observed (*continuous red lines*) number needed to treat per 1 year gained (NNT/y^+^) curves throughout the three trial and estimated NNT/y^+^ (*eNNT/y*^*+*^; *dotted red lines*) curves up to time 72 months. The arrows show the time at which the 50% of the cohort was in follow-up for PROVE-IT and LIFE trial and the 50% of the duration of the study for the HOPE trial

The MoT/y^+^ curves (Fig. 3) show that the number of months of treatment necessary to gain 1 year of event-free life decline rapidly during the first 24 months of the trial and slowly thereafter. The kinetics of this index in all the three studies is well represented by a negative power function whose adequacy is supported by the high regression coefficient values that were calculated on the whole available study data set (Table 1). The extreme difference in cost-benefit of the three different interventions applied to the three different populations clearly emerges: the ranking being 1:2:4 in PROVE-IT, HOPE and LIFE.

### The NNT/y^+^ curves

The NNT/y^+^ shows the number of subjects who need to be treated to gain 1 year of event-free time at any given time during the trial. This index rapidly decreases over the first 24 months; after which, its slope progressively approaches zero. The differences among the studies lie in the readiness of emergence of the effect (time at 200) and in the initial slope affecting the time lag necessary for the index to reach the same values. For example, in PROVE-IT, HOPE and LIFE trial values of 200 and 50 are reached at 6 and 13.3, 16 and 29.3 and 13 and 32.5 months, respectively. All the curves are well fitted by negative power functions with a high accuracy as shown by the regression coefficient values (Table 1). Again the projections at 6 years show large differences among the studies.

## Discussion

Our main finding is that the PISA method, by reconstructing from KM curves, the time-dependent relationship between real exposure and real benefit, allows a true, accurate and meaningful representation of the results of any RCT. Indeed, three apparently similar RCT emerge as very heterogeneous when dissected in the clinical (Time-Gain curves) and pharmaco-economic (MoT/y^+^ and NNT/y^+^) domain. We also unrevealed that the most relevant clinical and economic indices of any positive clinical trial follow a predictable kinetics, a property that facilitates data calculation and allows data modelling.

The need to improve the information generated by RCT, already presented more than 60 years ago [13] and recently prompted by authors particularly active in oncology [1, 6, 14–16], has emerged also for the limited reliability of risk ratio, and also NNT, in adequately representing the clinical significance of the eventually successful intervention. This integrated index is based on the assumption, seldom verified, of constancy throughout the study, does not take into consideration the kinetics (i.e. time-dependency) of the effect and implies the possibility to prevent an outcome, which for populations bearing a high risk for that event, is misleading. What is generated by a successful intervention is a gain in event-free time that is enjoyed by the whole cohort as a unit according to a specific kinetics. Any intervention, in fact, will have a different interaction with time depending on the mechanism/s it interferes with to slow down the progression of the disease. Unfortunately, CV prevention trials that are designed to accumulate a given number of events—in order for the risk ratio to achieve statistical significance—tend to shorten the study duration by increasing the sample size making it difficult to adequately take into account the time-dependent effects and eventually appreciate the differences among interventions.

The time gained by the cohort receiving the superior treatment is indeed a function of time, which can be precisely calculated as the difference between the areas of the KM incidence curves. This peculiar curve was already described as difference between restricted mean survival time (RMST) curves by Zhao et al. [17], but the peculiarity of its kinetics and its modelling has never been evaluated. These curves have the rather unexpected property of being smooth and to follow a consistent mathematical pattern, which can be accurately described by a second-order polynomial function. Clearly, the upraise (slope) of the time gain is not expected to continue indefinitely but only until approximately 50% of the subjects have developed the outcome, after which the curve will tend to plateau (as the number of subjects who can benefit from the treatment is progressively reduced). This pattern is shared by all CV prevention positive studies regardless of the type of intervention as shown in this analysis and also verified in other studies using antithrombotic or antidiabetic drugs. The first-order coefficient represents the linear and constant gain over time (as would be generated two KM parallel curves) and the second-order coefficient represent the time-dependent progression, i.e. the angle between the two KM curves. The different kinetics, particularly the onset, depends to some extent on the absolute level of risk of the population, but also on whether, and to what extent, the intervention also interferes with the clinical emergence (first-order coefficient) of the background disease, or mainly with its progression (second-order coefficient). In our analysis, the different kinetics of cholesterol-lowering (PROVE-IT) and blood pressure-lowering drugs (LIFE and HOPE) is evident both in qualitative and in quantitative terms. Interestingly, although the three interventions evaluated yielded comparable RR reductions (and comparable time gains at the end of the study), the time gain of the PROVE-IT at 2 years was 2.4-fold higher with respect to the other two studies (Table 1).

By using the PISA method, not only the effect of an intervention can be accurately described in the time domain, but also the exposure can be precisely measured throughout the study. From the incidence curves, we can calculate the complementary survival curves, which represent the true exposure of each cohort to the treatment. By doing this, we obtain two time-dependent indices (MoT/y^+^ and NNT/y^+^), which allow to appreciate how the cost effectiveness of the intervention changes with time. These curves also have the property of being smooth and to follow a kinetics that is very well described by a mathematical function. The major characteristics of these curves that reflect the cost normalized per unit of gain are that they display a rapid decline in the first 12–24 months and then slowly tend to approach a plateau. By describing objectively how the cost efficacy improves over time, these curves allow to compare the different interventions. The achievement of cost-efficacy level of 1000 MoT/y^+^ is reached after 6.3, 39.5 and > 72 months in PROVE-IT, HOPE and LIFE trials respectively. Whereas the broader primary composite outcome of the PROVE-IT in part justifies its quicker and greater cost effectiveness, the difference between HOPE and LIFE for the identical composite outcome is a fact to be taken into account in cost-efficacy analysis of the two different therapeutic strategies. Another relevant aspect that emerges from the inspection of the curves is that the cost efficacy of the HOPE tend to increase progressively (lower MoT/y^+^ values) with time. Therefore, the heterogeneity of the trials can be well appreciated both in absolute terms and also in the kinetics. Thanks to the curve fitting, it is possible to compare NNT at the same time (Table 1) and also to estimate how long would it take for an intervention to reach an NNT which is considered cost effective. For example, before the value of NNT/y^+^ goes below 50, it is necessary to wait 13.3, 29.3 and 32.5 months for PROVE-IT, HOPE and LIFE respectively. Due to the different kinetics of the curves, whereas at 2 years the NNT/y^+^ is similar for HOPE and LIFE the projections at 6 years indicate a two/fourfold difference among the studies. Clearly, this does not translate into a superiority of an intervention since the study design, outcomes and population were different, but would be a relevant information in case of studies sharing similar patient’s characteristics, similar design and identical outcomes. In addition, since the index is normalized for 1 year of event-free life, this allows an easy quantification of the real benefit; in economic terms, it is in fact possible to calculate the value of this unit, while this is more difficult when, as it is commonly done, the unit of gain is expressed as 1 event less, which again implies the concept that this event is really avoided, while it is more likely to be postponed. The novel index we here propose MoT/y^+^ in our opinion is particularly meaningful. It is based on a more accurate representation of the exposure (MoT), which taking simultaneously into consideration both the number of patients being treated and the duration of the treatment, allows a fair comparison among the studies also when having different durations. As evident from Table 1, while at study end the NNT/y^+^ of the three trials was similar, the corresponding values of MoT/y^+^ indicate a clear difference among the three interventions. In addition, due to its slower kinetics MoT/y^+^ allows a better discrimination in the late, and also economically more relevant, part of the study.

Another major asset of the PISA method is that it allows to estimate the results of any positive trial beyond its actual duration. Although we fully recognize the limitations inherent to the extrapolation of the data, we base our confidence on the reliability of the results on the following arguments. First, the time gain functions of cardiovascular prevention trials with durations up to 10 years (UKPDS-34, STENO-2) all share the same second-order polynomial pattern and within the same RCT this pattern is present both on compound and individual outcomes. Second, although the data fit is based only on a portion of the whole duration of the study (*t*_0_–*t*_50%_), the extrapolation beyond *t*_50%_ tend to closely represent the real data and this in the HOPE study holds true over a period of 27 months. Third, the extremely large sample size, characteristic of most CV prevention studies, increases the accuracy with which the effect is measured particularly in the early part of the study, on which the data fit is based.

The major limitation of the PISA method is that it does not allow to calculate the error of the estimates when performed on secondary data. Indeed, data at the patient level would be necessary. This would allow calculation of the 95% confidence intervals of the Kaplan-Meier curves and the parameters estimates using the 2 extreme pairs (97.5% active vs 2.5% control and 2.5% active vs 97.5% control) to produce the 95% confidence intervals. Of note, if the original Kaplan-Meier curves are presented with their confidence boundaries, it would be possible to calculate the error without accessing to patient-level data. Alternatively, provided that methods have been recently developed to calculate reliable confidence intervals from summary data [18], this limitation might be overcome.

In conclusion, the PISA method dissects the whole information enclosed in KM curves. Being simple and easily reproducible, it might improve survival data analysis by providing clear definition of trial results, allowing fair comparisons between similar RCT and performing accurate predictions beyond the trial duration.

## Supplementary information

**Additional file 1: Supplementary Figure 1.** Data extraction. The figure shows the accuracy of the data extraction using the UN-SCAN-IT Graph Digitalizer software on the Kaplan-Meier of PROVE-IT, LIFE and HOPE trials, respectively for 4p-MACE*, 3p-MACE and 3p-MACE outcomes. Point assignment was set at the mid line of upper and lower surface of the curve.

**Additional file 2: Supplementary Figure 2.** Glossary. Full glossary with definitions and mathematical formulae.

**Additional file 3: Supplementary Figure 3.** Time Gain observed and fitted curves from other major trials. Time Gain curves (*continuous line*) and fitted Time Gain curves *(f50%*; *dotted lines*) with the extrapolation beyond time at which less than 50% of the cohort was in follow-up (*indicated by arrows*) throughout the duration of the studies for various outcomes of some CV prevention trials: UKPDS-34, STENO-2, CIBIS-II, EMPA-REG OUTCOME, CANVAS, LEADER, SUSTAIN-6. With regards to CIBIS-II and SUSTAIN-6 trial (due to population in follow-up > 50% at the end of the trial) the fit and the extrapolation were performed at the end of the trial (*indicated by arrows*). The polynomial second order function obtained by performing curve fitting is displayed for each outcome along with the R^2^. a) UPKPDS-34, Death from any cause outcome; b) STENO-2, CV events outcome: a composite of cardiovascular disease events that included death from cardiovascular causes, nonfatal stroke, nonfatal myocardial infarction, coronary-artery bypass grafting, percutaneous coronary intervention or revascularization for peripheral atherosclerotic arterial disease, and amputation because of ischemia; c) CIBIS II: Death from any cause outcome; d) EMPA-REG OUTCOME, CV Death outcome; e) EMPA-REG OUTCOME: Heart Failure outcome, f) CANVAS: Death from any cause outcome; g) LEADER: 3p-MACE outcome, h) SUSTAIN-6: 3p-MACE outcome.

**Additional file 4.**

## Data Availability

The datasets used and/or analysed during the current study are available from the corresponding author on reasonable request. The original Kaplan-Meier Graphs are available in the PDF articles of the PROVE-IT [10], LIFE [11] and HOPE [12] trials**.**

## Abbreviations

RCT: Randomized clinical trials
HR: Hazard ratios
NNT: Number needed to treat
CV: Cardiovascular
KM: Kaplan-Meier
PISA: Pragmatic Interpretation of Survival Analysis
MoT: Months of treatment
