# Supplementary figures and images for: A novel method for interpreting survival analysis data: description and test on three major clinical trials on cardiovascular prevention
Source: Trials. 2020 Jun 26;21:578. doi: 10.1186/s13063-020-04511-y (PMC7318394; doi:10.1186/s13063-020-04511-y)

# PROVE-IT

4p-Mace\*

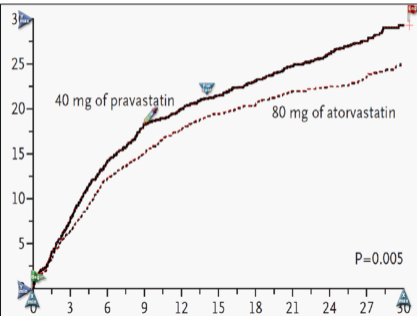

# LIFE

3p-Mace

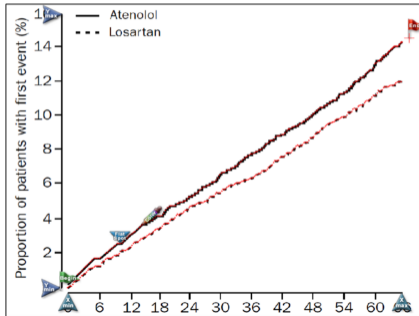

# HOPE

3p-Mace

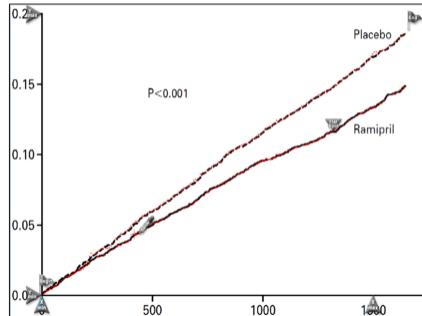

Supplement: Supplementary file 1 — Additional file 1: Supplementary Figure 1. Data extraction. The figure shows the accuracy of the data extraction using the UN-SCAN-IT Graph Digitalizer software on the Kaplan-Meier of PROVE-IT, LIFE and HOPE trials, respectively for 4p-MACE*, 3p-MACE and 3p-MACE outcomes. Point assignment was set at the mid line of upper and lower surface of the curve. [file 13063_2020_4511_MOESM1_ESM.pdf]
