# Supplementary material for: A novel method for interpreting survival analysis data: description and test on three major clinical trials on cardiovascular prevention
Source: Trials. 2020 Jun 26;21:578. doi: 10.1186/s13063-020-04511-y (PMC7318394; doi:10.1186/s13063-020-04511-y)
Supplement: Supplementary file 2 — Additional file 2: Supplementary Figure 2. Glossary. Full glossary with definitions and mathematical formulae. [file 13063_2020_4511_MOESM2_ESM.pdf]

Glossary

**Name**	**Description**	***f(x)***
**T**	Duration (months) of the study	
**iKM(t)**	Inverse Kaplan-Meier curve	
**KM(t)**	Kaplan-Meier curve	$t = \{t_0, t_1, t_2 \dots, T\}$<br>$KM(t) = 100 - iKM(t)$
**Time Lost**	Time course of the total units of time spent by the subjects after the event has occurred during the study follow-up normalized to 100 individuals	$\int_o^\tau iKM(t)dt$
**Time Gain**	Time course of the extra units of event-free time that are lived by 100 subjects in the active treatment arm (a) with respect to 100 in the control arm (c).<br>Positive values indicate a protective effect of the active treatment whereas negative values represent a harmful effect.	$\int_o^\tau iKMc(t) - iKMa(t)dt$
**$t_{50\%}$**	Time at which the population in follow-up falls below 50%.	
**Time Gain f50%**	Fitting of the Time Gain curve performed in the interval [0;$t_{50\%}$]. The fit is a polynomial second order function, forced to pass through the origin ($y = at^2 + bt$)	$Fit[0; t_{50\%}]\left\{\int_o^\tau iKMc(t) - iKMa(t)dt\right\}$
**MoT**	Months of treatment: time course of the exposure to the treatment expressed in months and normalized to 100 subjects	$\int_0^\tau Kma(t)dt$
**y^+**	Time Gain expressed in year	$\frac{\int_o^\tau iKMc(t) - iKMa(t)dt}{12}$
**MoT/y^+**	Months of treatment necessary to gain 1 year of event-free life as measured by the Time Gain curve	$\frac{\int_o^\tau KMa(t)\,dt}{\frac{\int_o^\tau iKMc(t) - iKMa(t)dt}{12}}$
**MoT/y^+f50%**	Months of treatment necessary to gain 1 year of event-free life as measured by the Time Gain f50% function	$\frac{\int_o^\tau KMa(t)\,dt}{\frac{\left(Fit[0; t_{50\%}]\left\{\int_o^\tau iKMc(t) - iKMa(t)dt\right\}\right)}{12}}$
**t_{6m}**	Time at which the *Time Gain Obs* = 6 months	
**eMoT/y^+**	Estimated MoT/y^+ obtained through the fitting of the MoT/y^+f50% curve performed in the interval [t_{6m}; T]. The fit is a power function ($y = at^b$)	$Fit[t_{6m}; T]\left\{\frac{\int_o^\tau KMa(t)\,dt}{\frac{\left(Fit[0; t_{50}]\left\{\int_o^\tau iKMc(t) - iKMa(t)dt\right\}\right)}{12}}\right\}$
**NNT**	Number needed to treat: time course of the subjects on treatment normalized to 100 subjects	$\frac{\int_o^\tau KMa(t)\,dt}{t}$
**NNT/y^+**	Number of patients who need to be treated to gain 1 year of event-free life as measured by the Time Gain curve	$\frac{\frac{\int_o^\tau KMa(t)\,dt}{t}}{\frac{\int_o^\tau iKMc(t) - iKMa(t)dt}{12}}$
**NNT/y^+f50%**	Number of patients who need to be treated to gain 1 year of event-free life as estimated through the Time Gain f50% function	$\frac{\frac{\int_o^\tau KMa(t)\,dt}{t}}{\frac{\left(Fit[0; t_{50\%}]\left\{\int_o^\tau iKMc(t) - iKMa(t)dt\right\}\right)}{12}}$
**eNNT/y^+**	Estimated NNT/y^+ obtained through the fitting of the NNT/y^+f50% curve performed in the interval [t_{6m}; T]. The fit is a power function ($y = at^b$)	$Fit[t_{6m}; T]\left\{\frac{\frac{\int_o^\tau KMa(t)\,dt}{t}}{\frac{\left(Fit[0; t_{50}]\left\{\int_o^\tau iKMc(t) - iKMa(t)dt\right\}\right)}{12}}\right\}$
