# Supplementary material for: A novel method for interpreting survival analysis data: description and test on three major clinical trials on cardiovascular prevention
Source: Trials. 2020 Jun 26;21:578. doi: 10.1186/s13063-020-04511-y (PMC7318394; doi:10.1186/s13063-020-04511-y)
Supplement: Supplementary file 3 — Additional file 3: Supplementary Figure 3. Time Gain observed and fitted curves from other major trials. Time Gain curves (continuous line) and fitted Time Gain curves (f50%; dotted lines) with the extrapolation beyond time at which less than 50% of the cohort was in follow-up (indicated by arrows) throughout the duration of the studies for various outcomes of some CV prevention trials: UKPDS-34, STENO-2, CIBIS-II, EMPA-REG OUTCOME, CANVAS, LEADER, SUSTAIN-6. With regards to CIBIS-II and SUSTAIN-6 trial (due to population in follow-up > 50% at the end of the trial) the fit and the extrapolation were performed at the end of the trial (indicated by arrows). The polynomial second order function obtained by performing curve fitting is displayed for each outcome along with the R2. a) UPKPDS-34, Death from any cause outcome; b) STENO-2, CV events outcome: a composite of cardiovascular disease events that included death from cardiovascular causes, nonfatal stroke, nonfatal myocardial infarction, coronary-artery bypass grafting, percutaneous coronary intervention or revascularization for peripheral atherosclerotic arterial disease, and amputation because of ischemia; c) CIBIS II: Death from any cause outcome; d) EMPA-REG OUTCOME, CV Death outcome; e) EMPA-REG OUTCOME: Heart Failure outcome, f) CANVAS: Death from any cause outcome; g) LEADER: 3p-MACE outcome, h) SUSTAIN-6: 3p-MACE outcome. [file 13063_2020_4511_MOESM3_ESM.pdf]

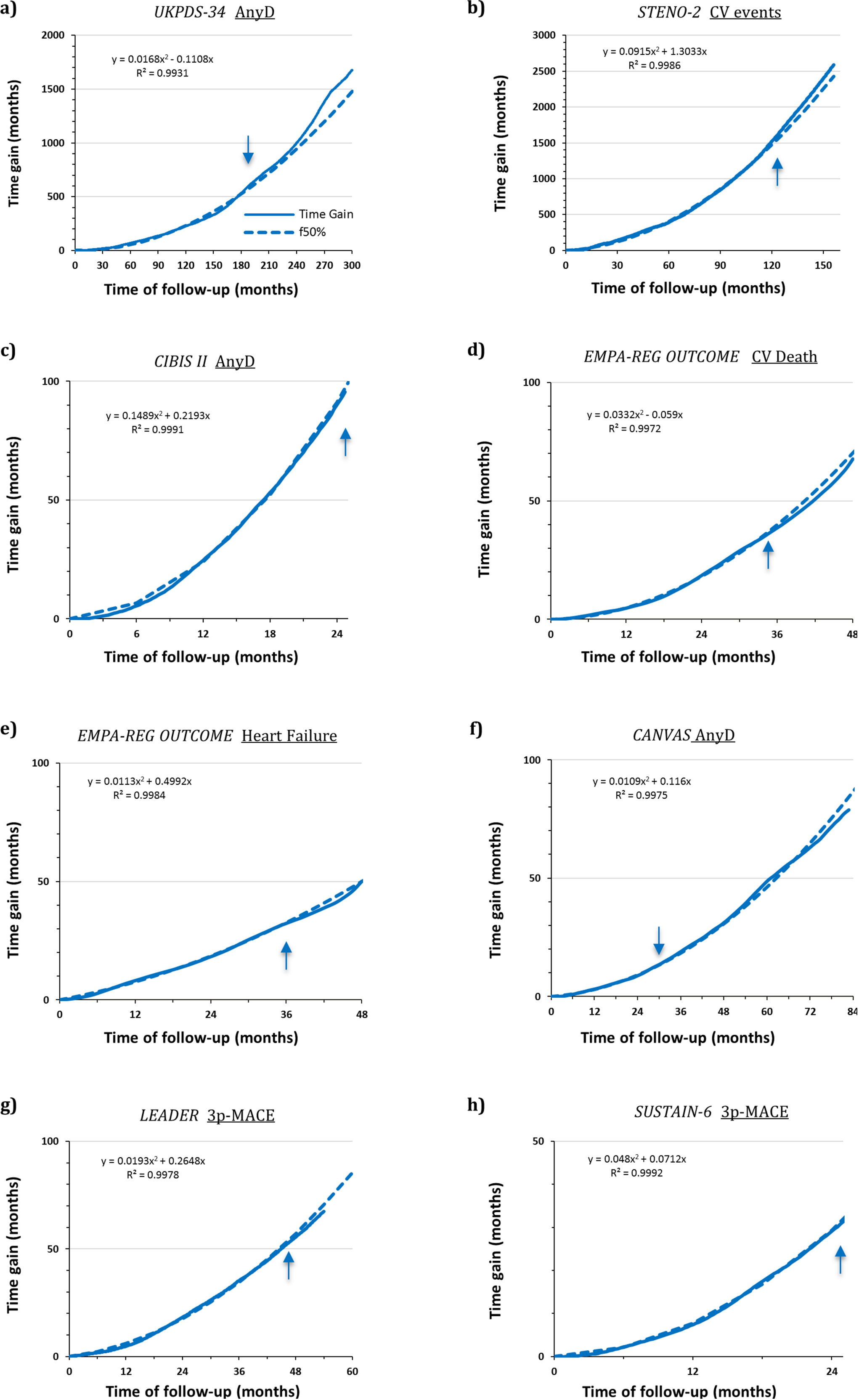
a)
UKPDS-34 AnyD
y = 0.0168x2 - 0.1108x
R² = 0.9931
Time Gain
f50%
Time gain (months)
Time of follow-up (months)
0
500
1000
1500
2000
0 30 60 90 120 150 180 210 240 270 300
b)
STENO-2 CV events
y = 0.0915x2 + 1.3033x
R² = 0.9986
Time gain (months)
Time of follow-up (months)
0
500
1000
1500
2000
2500
3000
0 30 60 90 120 150
c)
CIBIS II AnyD
y = 0.1489x2 + 0.2193x
R² = 0.9991
Time gain (months)
Time of follow-up (months)
0
50
100
0 6 12 18 24
d)
EMPA-REG OUTCOME CV Death
y = 0.0332x2 - 0.059x
R² = 0.9972
Time gain (months)
Time of follow-up (months)
0
50
100
0 12 24 36 48
e)
EMPA-REG OUTCOME Heart Failure
y = 0.0113x2 + 0.4992x
R² = 0.9984
Time gain (months)
Time of follow-up (months)
0
50
100
0 12 24 36 48
f)
CANVAS AnyD
y = 0.0109x2 + 0.116x
R² = 0.9975
Time gain (months)
Time of follow-up (months)
0
50
100
0 12 24 36 48 60 72 84
g)
LEADER 3p-MACE
y = 0.0193x2 + 0.2648x
R² = 0.9978
Time gain (months)
Time of follow-up (months)
0
50
100
0 12 24 36 48 60
h)
SUSTAIN-6 3p-MACE
y = 0.048x2 + 0.0712x
R² = 0.9992
Time gain (months)
Time of follow-up (months)
0
50
0 12 24
